# High incidence and low case detection rate among contacts of tuberculosis cases in Shanghai, China

**DOI:** 10.1186/s12879-019-3942-2

**Published:** 2019-04-11

**Authors:** Juntao Guo, Meixia Yang, Zheyuan Wu, Xin Shen, Yuanhui Wang, Genming Zhao

**Affiliations:** 1 Institute of Tuberculosis and HIV/AIDS Control and Prevention, Shanghai Municipal Center for Disease Control and Prevention, Shanghai, China; 2 Department of Tuberculosis and HIV/AIDS Control and Prevention, Xuhui District Center for Disease Control and Prevention, Shanghai, China; 3 Department of Epidemiology, School of Public Health, Fudan University, Shanghai, China

**Keywords:** Tuberculosis, Index case, Contact investigation, Disease density

## Abstract

**Background:**

To assess the effect of a contact investigation strategy by assessing the incidence of tuberculosis and the case detection rate among contacts of tuberculosis patients.

**Methods:**

The pulmonary tuberculosis incidence among contacts was determined retrospectively from a tuberculosis information management system. For each detection method (symptom examination only, symptom examination plus chest radiography or other alternatives), the detection rate of pulmonary tuberculosis patients among contacts was derived from contact investigation form records.

**Results:**

Sixty-nine cases of pulmonary tuberculosis were identified among a total of 8137 contacts after an average follow-up of 2.6 years (range: 0.25–5.25) during the period from 2010 to 2014. The incidence density was 329/100,000 person-years (PYs), and the 95% confidence interval (CI) was 256–419/100,000 PYs, which was significantly higher than the notification rate during the same period in the general population (29–30/100,000 PYs). The incidence density was higher (*p* < 0.0001) among male contacts (462/100,000 PYs) than among female contacts (236/100,000 PYs). The incidence density did not differ (*p* > 0.05) between contacts whose index case was sputum smear positive and those whose index case was sputum smear negative. Contacts who were biologically related family of the index cases exhibited a higher (*p* < 0.05) incidence density (475/100,000 PYs) than other contacts (281/100,000 PYs). Fifteen of the 69 incident cases were found through contact investigation, corresponding to a case detection rate via contact investigation of 22% (95% CI: 13–33%). The relevance ratio was 288/100,000 (12/4163) by both chest radiography and symptom survey, which was significantly higher than the rate detected by symptom survey alone, of 57/100,000 (2/3486), *p* = 0.028. The cumulative incidence in the contacts was 761/100,000 (62/8137) within 3 years from the time that the index cases were diagnosed with pulmonary tuberculosis, which was higher than the incidence rate of 210/100,000 (7/3328) recorded after 3 years (*p* < 0.001).

**Conclusions:**

The contacts were at higher risk of pulmonary tuberculosis than the general population; however, only approximately 22% of the incident cases could be detected through contact investigation. Therefore, the contact investigation strategy must be improved for better detection of potential pulmonary tuberculosis cases.

## Background

China is one of the high Tuberculosis (TB) burden countries, ranked 2nd in terms of the absolute number of incident cases even at present. However, over the past 2 decades, China has made tremendous efforts in TB control and prevention: by 2004, the Government had implemented the DOTs strategy along with the built of world’s largest internet-based communicable-disease reporting system; by 2010, China has reduced TB prevalence and mortality rate by half, which is 5 years ahead of the target year of the Millennium Development Goals (MDGs) set by United Nation [1, 2]. Besides disease reporting and DOTs strategy focused on active cases, contact investigation is another important part of TB control and prevention. Contacts of pulmonary TB patients are a group at high risk of developing TB, and individuals who have contact with smear-positive TB patients present even higher risk [3]. Contact investigation and active surveillance of smear-positive TB cases are two important methods for identifying TB patients and preventing the spread of *Mycobacterium tuberculosis* among the general population. According to the World Health Organization’s (WHO’s) recommendations for middle- and low-income countries [4, 5], household or close contacts of infectious TB should be screened to find active TB cases, whereas emphasis should focus on index case who is sputum smear-positive, MDR/XDR TB, PLHIV and child < 5 years of age. In addition, a PLHIV and a child < 5 years of age who is a household or close contact of TB is encourage to treat for LTBI. As a TB high burden country with, limited funding for its controlthe contact investigation strategy applied in China mainly targets on sputum smear-positive TB index case in order to identify possible active cases.. Therefore, our contact screening methods are quite different from those of the United States and other developed countries [6]. It has been a national strategy in China to conduct active tracing and provide free screening tests to close contacts (including household and other close contacts) of smear-positive TB patients since 2006 [7]. In 2006, the former Bureau of Disease Prevention and Control under the Ministry of Health (BoDP&C-Moh) issued a document requiring TB control institutions at all levels to implement this strategy. Over the subsequent years, this strategy has been widely adopted and implemented by TB control institutions across the nation. This strategy requires contact investigation of all smear-positive TB patients, in which the contact is first asked whether any suspected TB symptoms are present, and further examinations are then performed on contacts with suspected TB symptoms, individuals with active TB infection will go under further treatment [7]. From 2006 to 2012, Shanghai adopted the national strategy for TB contact screening issued by BoDP&C-Moh. However, we found that very few cases could be identified by this screening strategy. Given that the annual incidence of TB in Shanghai has decreased to 22–26/100,000 (which is lower than in other regions), and funding for TB control has become more abundant, the national strategy has since been modified to better suit local needs. During the years 2013 to 2014, the former BoDP&C-Moh of Shanghai performed a pilot study in 4 districts, which expanded the tracing of contacts of smear-positive TB patients to the tracing of contacts of all active TB patients (both smear-positive and smear-negative TB patients) and provided free chest radiographys to every contact investigated. It is our hope that through these improvements, we will be able to use contact screening to identify more active TB patients among contacts. When the identification rate of active patients among contacts has reached a satisfactory level, we will consider shifting the focus from finding TB cases to the detection and curing of latent TB infections. In the current study, we performed a retrospective analysis of the data collected from the 4 pilot districts in Shanghai during the period from 2013 to 2014 and the 3 years (2010 to 2012) before these modifications were implemented. To assess the effect of the contact investigation strategy and provide data support for improving this strategy, we calculated the incidence and case detection rates of TB patients among all the contacts and investigated their association with population characteristics such as sex and age.

## Methods

### Study sites and participants

The study participants included individuals who had contact with TB patients who were diagnosed between January 1, 2010 and December 31, 2014 in the Xuhui, Changning, Minhang and Songjiang districts of Shanghai. In this analysis, the index cases were defined as TB patients, and contacts were defined as individuals who had direct contact with an index case, including family members, colleagues, classmates and roommates.

### Contact investigation

**Fig. 1:**
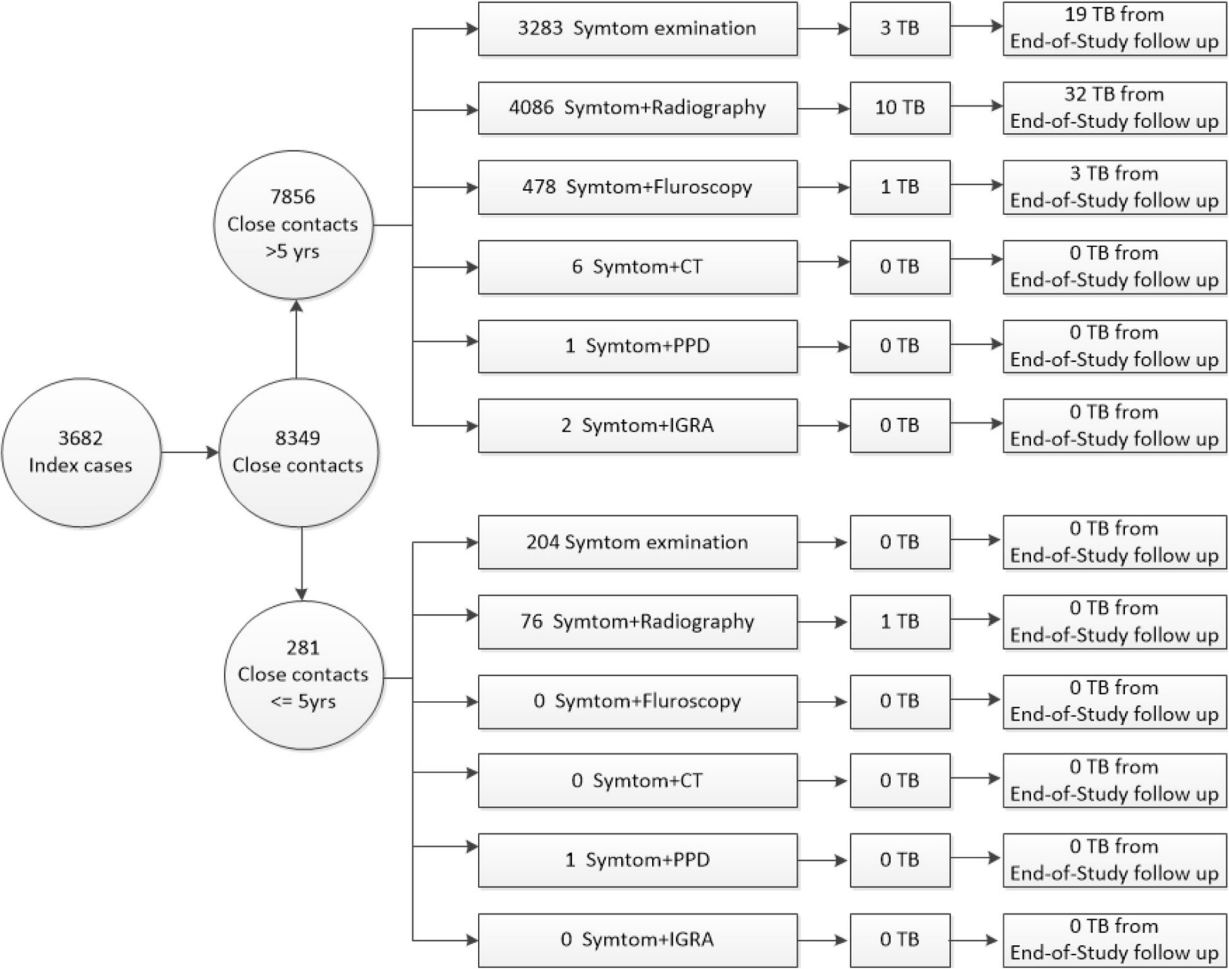
Flow-chart of the contact investigation with active pulmonary TB stratified by age and contact investigation methods

During 2010 to 2012, the contact investigation strategy implemented in the four districts was the same as the national strategy, which involved only the tracing of contacts of smear-positive index cases. As required by Chinese Infectious Disease Control and Prevention law, designated TB hospitals are responsible for reporting every TB patient diagnosed to the National TB Report System, with detailed information on TB disease and other complementary data, including individual identification information, names, and home addresses. Doctors from community health centers can log onto this report system daily to determine whether any new sputum smear- positive pulmonary TB cases have been reported. If a new case is reported, then the community doctor scheduled a home visit with the patient within 3 days and obtained a list of names of individuals with whom the patient has had direct contact within congregated settings (mainly concerning poor air ventilation). The names on this list are the contacts of this index case. Community doctors then implement a series of contact investigations within a 30-day period from the diagnosis of the index patient. The contact investigations are performed through home visits with each contact. Suspected TB symptoms include 1 or more of the following symptoms: 1. coughing for more than 2 weeks; 2. hemoptysis (coughing up blood); and 3. coughing and expectoration for > 1 week, but < 2 weeks, accompanied by fever in the afternoon, night sweats, loss of appetite, fatigue or weight loss. If there are no suspected TB symptoms, the contact investigation ends at this point. If any suspected symptoms are found, the contact is provided with a free chest radiography and other tests in an appointed hospital. For children under 15 years of age, the Tuberculin Skin Test (purified protein derivative, PPD) is performed instead of a chest radiography [7–9]. During 2013 to 2014, four districts implemented the modified strategy involving the tracing of contacts of all active pulmonary TB cases. Under this strategy, community doctors visit all contacts for the investigation of symptoms and require all contacts to visit an appointed hospital for chest X-ray screening or other equivalent tests, regardless of any suspected symptoms. For children under 15 years of age, instead of chest radiography, PPD is performed, regardless of their symptoms. If the results of the screenings performed during contact investigation are not normal, the contact is referred to a designated TB hospital to undergo further evaluations and a series of confirmatory tests to determine whether the individual is infected with TB (Fig. 1).

All TB patients included in our study were diagnosed by clinical doctors specialized in TB diagnosis and treatment from designated TB hospitals according to national guidelines [10]. According to the microscopy smear results, TB is classified as smear-positive or smear-negative TB. Similarly, the result of TB mycobacterial culture classifies TB as culture-positive or culture-negative TB. TB cases with both smear-negative and culture-negative results are classified as bacterial-negative cases, whereas cases with either or both positive results are classified as bacterial-positive cases. Bacterial-positive TB disease is diagnosed based on evidence from the microscopy smear and mycobacterial culture results. Bacterial-negative TB disease is diagnosed under the following circumstances (satisfying any 3 of conditions 1–6 simultaneously or either circumstance 7 or 8): 1. symptoms and radiographic signs suggestive of TB; 2. effectiveness of diagnostic anti-TB therapy; 3. other lung diseases are ruled out clinically; 4. a strong positive result for PPD (5TU) and a positive result for serum antibodies; 5. a positive result from polymerase chain reaction (PCR) and gene probe assays of the sputum sample; 6. pathological confirmation of extrapulmonary TB; 7. detection of acid-fast bacillus (AFB) in a bronchoalveolar lavage fluid (BALF) sample; and 8. pathological confirmation of bronchial or pulmonary TB [10].

A Contact Registration Form was kept for each contact, with their personal identification number (ID), name, demographic information, examinations performed, and results.

### Data extraction

The National TB Report System was used as the basis for determining disease status among the contacts of TB patients. We believe that this system is reliable in the tracing of contacts of TB patients for the following reasons: first, this system covers all designated institutions in Shanghai where TB patients seek care; second, the Chinese law of Infectious Disease Control and Prevention requires that all suspected and confirmed cases of TB disease must be reported within 24 h through this system; and third, Shanghai has a relatively sound regimen for checking under-reporting of infectious diseases. All hospitals must conduct a self-check every month for under-reporting; the district Centers for Disease Control and Prevention (CDC) perform a full-coverage investigation of under-reporting on all hospitals quarterly; and the CDC of Shanghai conducts a spot check on selected hospitals once a year. This regimen keeps under-reporting of infectious diseases at a very low level.

Since the study period ranged from January 1, 2010 to December 31, 2014, the tracing of the disease status of contacts using the National TB Report System began later in April of 2015. When searching for cases in the system, the time frame was set to January 1, 2010 to March 31, 2015, using the Chinese personal ID as a unique matching identifier to match the cases within the system with contacts from the Contact Registration Form. To further reduce under-reporting bias, contacts that were not found in the system were approached (either by home visit or through a phone call) by community doctors to assess their TB disease status within the same time period.

### Statistical analysis

The demographic characteristics of the contacts, including their sex, age, and relationship with the index case, were included in the analysis. The relationship with the index case was categorized as non-relative, wife/husband, parent/child, sibling or other relative.

The data were maintained in Excel 2007 and analyzed with the statistical software Stata, version 12.0. (http://www.stata.com/). The chi-square test was used to compare the rate of TB among contacts, and the Poisson test was used to compare TB disease density. A significance level of 0.05 was employed for all tests.

### Ethical approval

Ethical approval for this study was obtained from the Institutional Review Board of the Shanghai Municipal Center for Disease Control and Prevention. Lists of potential participants (including index cases and contacts) were emailed to each study coordinator in the four districts. Trained community doctors then called each participant in their community to obtain their verbal consent. For children under the age of 16, consent was obtained from their parents or legal guardian. For those who could not be reached by telephone, a home visit was conducted by community doctors to obtain their verbal consent. The study coordinator from each district then compiled and returned the list of individuals who gave their consent, along with their own statement and signatures to prove its authenticity. The name and identification number were removed in the data analysis step to preserve the confidentiality and anonymity of the participants from whom data were collected.

## Results

### Demographic information of the study participants

In total, 8349 contacts of 3682 index cases were included. After the exclusion of 212 participants with missing key information (i.e. index cases’ information, time of examination), 8137 contacts remained in the final analysis. On average, 2.2 contacts were analyzed per index case (range, 1–24 contacts), with a median age of 38.5 yrs. (SD = 18.2 yrs.; range, 1 to 98 years). Male contacts accounted for 46.6% of the participants. The average follow-up period was 2.6 years (SD = 1.4, range = 0.25–5.25).

### Incidence of TB

Sixty-nine cases of TB were found among the 8137 contacts, with a cumulative incidence of 848/100,000 over 5 years (95% CI: 660–1071). Altogether, 20,991 PYs were observed, with a TB incidence density of 329/100,000 PYs (95% CI: 256–416). Of the 69 TB cases among the contacts, 32 were smear-positive cases, 35 were smear-negative cases, and the remaining 2 cases involved children under 6 years old (both with BCG vaccination history) (data not shown in the tables). The incidence density of smear-positive TB was 153/100,000 PYs (95% CI: 105–216), and the incidence density of smear-negative TB was 117/100,000 PYs (95% CI: 117–233).

Bivariate analysis revealed that men (1160/100,000) exhibited a higher incidence of TB disease than women (575/100,000, *p* = 0.005) (Table 1). The contacts of smear-positive index cases presented a similar incidence of acquiring TB disease to the contacts of smear-negative index cases (840/100,000 vs. 870/100,000, *p* = 0.172) (Table 1). The biologically related family members of index cases exhibited a significantly higher incidence of TB than other contacts (1152/100,000 vs. 667/100,000, *p* = 0.021) (Table 1). Males and biologically related family members presented a higher risk of acquiring TB than did females and individuals with other relationships (*p* < 0.009 and *p* < 0.031, respectively) (Table 1). The contacts of smear-positive and smear-negative index cases showed a similar incidence density following adjustment (*p* = 0.217).

**Table 1:** Incidence and correlates of TB disease among 8137 contacts in the Xuhui, Changning, Minhang and Songjiang districts of Shanghai from 2010 to 2014

	No. of contacts	Observed pyrs	No. of TB disease cases	Cumulative incidence /100,000	95% CI	P	Incidence density /100,000 pyrs	Adjusted incidence density /100,000 pyrs^b^	95% CI	P
	8137	20,991	69	848	660–1071	–	329	–	–	–
Sex										
male	3792	9768	44	1160	844–1555	0.005	450	462	336–616	0.009
female	4345	11,223	25	575	373–848		223	236	158–350	
Age (years)										
1–9	436	1103	2	459	56–1647	0.195	181	184	22–653	0.18
10–19	529	1402	7	1323	534–2707		499	469	201–1026	
20–29	1992	5099	25	1255	814–1847		490	490	318–723	
30–39	1371	3592	6	438	161–950		167	161	61–363	
40–49	1544	4033	16	1036	593–1677		397	402	227–643	
50–59	1176	3023	8	680	294–1336		265	272	114–521	
60–69	635	1587	2	315	38–1133		126	160	39–551	
70–79	309	819	2	647	78–2318		244	450	133–1246	
80–89	133	310	1	752	19–4118		323	542	78–2318	
≥ 90	12	23	0	0	–		0	0	–	
Type of index case										
Smear+	5953	16,127	50	840	624–1106	0.892	310	327	246–430	0.217
Smear-	2184	4864	19	870	525–1355		391	456	284–684	
Bacterial+	7527	19,870	67	890	690–1129	0.172	337	348	270–439	1.000
Bacterial-	610	1121	2	328	40–1179		178	239	55–780	
Relationship with index case										
None-relatives	1175	2915	9	766	351–1449	0.139	309	180	56–400	0.008
Wife/husband	1561	4057	12	769	398–1339		296	283	153–516	
Parents/children	2728	7267	30	1100	743–1566		413	487	336–669	
Other relatives	2364	5920	13	550	293–939		220	205	105–354	
sibling	309	832	5	1618	527–3736		601	567	195–1397	
Biologically related family ^a^	3037	8099	35	1152	804–1599	0.021	432	475	332,643	0.031
Other relationship	5100	12,892	34	667	462–930		264	281	196–386	

^a^Biologically related family: parents/children and siblings

^b^Incidence density adjusted by age and gender

Multivariate logistic regression including gender, age group, relationship with index case (biologically related or not) and whether index case was bacterially confirmed as independent variables and disease outcome as dependent variable. A similar trend remained as bivariate analysis (Table 2). Males and biologically related family members still presented a higher risk of acquiring TB than did females and individuals with other relationships (OR = 1.93, *p* = 0.01 and OR = 1.91, p = 0.01, respectively). Contacts of smear-positive and smear-negative index cases still showed a similar incidence density (OR = 2.89, *p* = 0.14).

**Table 2:** Multivariate analysis on risk factors of TB among TB contacts by demographics and type of contacts

Factors	Odds Ratio	Std. Err.	z	P > z	95% CI	
Male vs. Female	1.93	0.49	2.59	0.01	1.17	3.17
Bacterial positive index case vs. negative cases	2.89	2.08	1.47	0.14	0.71	11.84
Biological related family vs. non-biological	1.91	0.478	2.58	0.01	1.17	3.12
Age group (years)						
10–19	3.10	2.49	1.4	0.16	0.64	15.01
20–29	3.48	2.58	1.68	0.09	0.81	14.90
30–39	1.12	0.92	0.14	0.89	0.22	5.59
40–49	2.55	1.92	1.24	0.21	0.58	11.16
50–59	1.59	1.26	0.59	0.56	0.34	7.55
60–69	0.87	0.87	−0.14	0.89	0.12	6.22
70–79	2.12	2.14	0.74	0.46	0.29	15.33
80–89	2.42	2.99	0.72	0.47	0.22	27.21
≥ 90	1	(no TB case)				
_cons	0.0007467	0.0007688	−6.99	0	0.0000992	0.005618

### Characteristics of the 69 TB cases among the contacts

Among all 69 cases of TB found in this study, 15 were identified through contact investigation, and the other 54 cases were identified in other ways during the follow-up period. The detection rate through contact investigation was 22% (15/69) (95% CI: 13–33%). Among the 32 smear-positive cases identified, 9 were found through contact investigation, with a detection rate of 28% (9/32) (95% CI: 14–47%). One case in a child who did not undergo sputum examination was identified during contact investigation.

All 8137 contacts were evaluated through contact investigation within 1 month of the diagnosis of the index cases. A total of 4163 contacts underwent both symptom examination and chest radiography; 478 underwent symptom examination and fluoroscopy; 6 underwent symptom examination and chest CT; 2 underwent symptom examination and the PPD test; and 2 underwent symptom examination and interferon-γ release assays (IGRAs) (Table 3). The remaining patient underwent symptom examination only. The chi-square test revealed a significantly higher incidence of TB disease among contacts who underwent both symptom examination and chest radiography than among those who underwent symptom examination only (288/100,000 vs. 57/100,000, *p* = 0.028). The TB detection rates associated with different investigation methods are listed in Table 3.

**Table 3:** Incidence of TB disease and detection rates for different screening methods among TB contacts

Screening methods	No. of contacts	TB cases from contact investigation	rate/100,000 (95% CI)	P#	TB cases not detected by contact investigation^a^	Total TB cases^b^	Detection rate%^c^	P
CT+ symptoms	6	0	–		0	–	–	–
PPD+ symptoms	2	0	–		0	–	–	–
IGRA+ symptoms	2	0	–		0	–	–	–
Symptoms	3486	2	57 (7–207)	0.028	19	21	9.5 (2/21)	0.122
chest radiograph+ symptoms	4163	12	288 (149–503)		32	44	27.3 (12/44)	
chest fluoroscopy + symptoms	478	1	209 (5–1160)		3	4	25.0	
Total	8137	15	184 (103–304)		54	69	21.7	

# Comparison of detection rates by symptom examination only and symptom examination plus chest radiography or other alternatives

^a^TB cases not detected by contact investigation: cases not identified by contact investigation but reported as TB cases in the National TB Report System between January 1, 2010 and March 31, 2015

^b^Total TB cases: cases identified by contact investigation and cases not identified by contact investigation but reported as TB cases in the National TB Report System between January 1, 2010 and March 31, 2015

^c^Detection rate = number of TB cases among contacts determined through contact investigation/total number of TB cases × 100%

Among the 69 cases of TB identified in this study, 15 (22%) were diagnosed within 1 month of the contact investigation, and another 20 were diagnosed within 1 year through medical treatment or physical check-up. The cumulative incidence of TB disease over 1 year was 430/100,000 (35/8137), with a disease density of 464/100,000 person-years (Table 4). Altogether, 62 cases were diagnosed within 3 years, and the remaining 7 cases were diagnosed within 5 years, with cumulative TB disease incidences of 761/100,000 (62/8137) and 210/10,000 (7/3328) for diagnosis within 3 years and 5 years, respectively. The risk of TB disease within 3 years was significantly higher than the risk after 3 years (*p* < 0.001) (Table 4).

**Table 4:** Frequency and incidence of TB disease by time among 8137 contacts in the Xuhui, Changning, Minhang and Songjiang districts of Shanghai from 2010 to 2014

Follow-up time (year)	No. of contacts	Observed pys	TB cases	Cumulative incidence /100,000	95% CI	Incidence density /100,000 pys	95% CI
0–1	8137	7538	35	430	300–598	464	323–646
1–2	6599	5844	13	197	105–337	222	118–380
2–3	4945	4213	14	283	155–475	332	182–558
3–4	3328	2514	3	90	19–263	119	25–349
4–5	1638	866	4	244	67–624	462	126–118
5–5.25	146	16	0	0	–	0	–
Total	8137	20,991	69	848	660–1071	329	256–416

## Discussion

### Characteristics of TB disease among contacts in Shanghai

This retrospective study revealed a high incidence of TB disease (848/100,000) among contacts of TB patients over an average of 2.6 years of follow-up. This rate is approximately 30 times higher than that in the general population (29.6/100,000) of Shanghai (from the Shanghai TB Report System, not published). This result (69/8137) is consistent with data reported by some researchers from Shandong, China and in California and Kenya [11–13]. However, many researchers from other countries have reported a higher incidence of TB disease among contacts: researchers from the US reported a rate ranging from 1.1–2.2% [14–17]; the UK reported a rate ranging from 2.9–5.9% [18–21]; and data from middle- and low-income countries (i.e., Turkey, Gambia, Iran and Thailand) revealed a rate ranging from 1.4–14.5% [22–25]. These higher rates reported in most studies were likely because most of the index cases included in their studies were smear-positive TB patients, and some studies even restricted contacts to close contacts or contacts within family. In contrast, we included contacts of both smear-positive and smear-negative TB patients and did not restrict contacts into specific groups.

The TB disease surveillance data revealed an increasing trend of TB disease among different age groups in Shanghai. We failed to identify such an association in the present study. This result is consistent with previous studies showing no significant difference among different age groups [26–29]. Nevertheless, a higher TB disease incidence density was found among individuals with ages of 20–29 years, who exhibited a disease incidence approximately 40 times that of the general population, which indicates the importance of implementing active contact investigation among this age group. In addition, this study showed that male contacts exhibited twice the risk of acquiring TB disease as female contacts. This result is consistent with data reported from the general population in Shanghai.

### Limitations of the current contact investigation strategy

Although current guidelines require contact investigation of all contacts experiencing direct contact with the index case, including family members, colleagues, classmates and roommates, the average number of contacts for each index case was only 2.3 in this study. This result is consistent with some other studies from this country [30, 31] and primarily results from a fear of being stigmatized or shunned by others, as many index cases do not want others to know they have TB and omit the names of colleagues, friends, classmates or even relatives from the contact list. Thus, the contacts who underwent contact investigation were mainly family members who lived under the same roof and were mostly the index cases’ biological family members. Future contact investigations may require community doctors to educate TB patients on the importance of contact investigation and simultaneously educate the general public with accurate information about TB to reduce the stigmatization toward TB patients. In addition, a lack of detailed contact information (i.e., the grade of the contact) is one of the limitations of the current contact investigation strategy. We were unable to collect accurate contact information years after the original contact investigation because of the retrospective nature of this study. Future studies must include the grade of the contact and ways to reach out to the contact to further quantify the disease risk under each circumstance and guide the contact investigation with better evidence. The detection rate through contact investigation in this study was only 22% (15/69), with the remaining TB disease cases being diagnosed through other means, including individual physical check-ups. This low detection rate revealed that the current strategy of contact investigation may lack effectiveness in identifying TB cases among the contacts of TB patients. This is mainly because the current contact investigation strategy restricts the screening of contacts to within 1 month from the index case’s diagnosis, which leaves out patients with disease onset after 1 month. Among all 69 TB cases diagnosed within contacts, 46% were diagnosed within 3 years of their index case’s diagnosis. Researchers from other countries have also indicated a high detection rate of TB among contacts within 3 years of diagnosing the index case [32]. A meta-analysis using data from 77 studies revealed that in middle- and low-income countries, contacts exposed to index cases exhibit the highest incidence of TB disease in the first year, with a rate of 1500/100,000, followed by a rate of 1000/100,000 in the 3rd year. This rate starts to decline in the 4th year, falling to approximately 500/100,000 [32]. Indeed, the follow-up period of a TB contact investigation is usually 2 years in the UK, the US, Japan, Taiwan, and China [33–35]. All these data suggest that annual follow-up may be necessary until the end of the 3rd year if healthcare resources allow.

The contact investigation strategy of performing chest radiography or fluoroscopy only on contacts with suspected TB symptoms detected 9.5% (2/21) of the TB cases. The detection rate was lower than that reported in other studies. For example, a study from Turkey indicated that a combined strategy of symptom assessment, chest X-ray evaluation and TB skin PPD testing detected 41.4% of TB cases among contacts [26]. This finding suggests that a combined screening strategy may increase the TB detection rate among contacts. Another study on family contacts of smear-positive TB cases from China found that only 50% of TB cases among contacts had suspected TB symptoms [36]. The results from this study indicate that performing chest radiography or fluoroscopy only on contacts with TB symptoms is not adequate since a large proportion of contacts who actually have TB may be asymptomatic. The contact investigation strategy applied from 2013 to 2014, in which chest radiography was added, increased the detection rate from the initial rate of 9.5 to 27%. Hence, performing chest radiography or the equivalent on every contact, instead of only symptomatic contacts, can increase the detection rate of TB cases among this population. Studies involving TB contact investigations from many countries have defined index cases as only smear-positive TB patients; however, the results from this study indicated that smear-negative TB patients also have a high risk of developing TB disease (not significantly different from the contacts of smear-positive TB patients). In addition, distinguishing between index cases and contact TB cases is sometimes difficult since it is quite possible that a smear-negative index case diagnosed earlier was actually infected through contact with a non-symptomatic case. As a result, contact investigation of only smear-positive index cases may not be adequate to detect non-symptomatic TB cases among contacts. The same conclusion was drawn by researchers from Arkansas, US [37]. Therefore, we believe that performing contact investigation among the contacts of all TB patients is beneficial for TB tracing among this population.

According to the contact investigation strategy recommended by the WHO for middle- and low-income countries, clinical evaluation of household and close contacts for active TB is a priority for TB disease control [4]. The Chinese strategy for TB contact investigation is aimed at identifying active cases of TB, which complies with the strategy recommended by the WHO. Nevertheless, the current screening methods adopted in China only identify a small percentage of disease patients, partly because chest radiography (or the equivalent) is administered only to contacts with suspected symptoms. Identifying active TB patients is a crucial step in TB control; however, identifying and treating latent TB are also an important strategy for decreasing TB incidence among the general population. With limited funding from the government, it is difficult to test and treat the large population with latent TB infection. Future endeavors may focus on finding a cost-effective way to test for latent infection in a contact investigation and may possibly treat LTBI among high risk groups such as child contact and PLHIV. We suggest a contact investigation strategy fully based on the WHO recommendations when resources are permitted.

Based on the results from this study, index case for contact investigation should be defined as both smear-positive and smear-negative active cases since index case may not necessarily be the source case. In terms of follow up period, longer period of follow up (at least 2 years) is suggested in order to identify possible incident cases. This is also consistent with the statement made by WHO that the risk of developing TB is increased for 1–2 years if infected through contact investigation. In addition, combine symptom examination with radiography will increase the case detection rate among the remaining 50% active cases without TB symptoms. Nevertheless, there is still a large gap between China and other developed countries. In the United States or other European countries, more expensive screening methods, such as IGRAs, are used in contact investigations, and latent TB infections found through the screening process also receive treatment. For a developing country, our current focus is still on identifying and treating active TB patients. We believe that testing and treating latent TB infection will be the next step.

IGRA or TST might be better alternatives in developed countries with low incidence, but can be very difficult to achieve under current circumstance in China because IGRA test is provided by TB designated hospitals as out-of-pocket service at a high price that cannot be reimbursed through government health insurance or other specific reimbursement plans. As regard to TST, it wasn’t proposed for two reasons. First, TST requires two hospital visits for each contact, and the timing of the second visit is restricted to within 48–72 h, which is difficult to achieve in reality. Second, because of the high percentage of BCG vaccinations among the Chinese population, the interpretation of TST results can be difficult since a considerable percentage of false positive will emerge. This situation is rather different from country low incidence country (i.e. the United States) where majority did not receive BCG vaccination.

## Conclusion

Contacts of TB patients exhibited a higher risk of acquiring TB disease than the general population in Shanghai. The modified contact investigation strategy applied during 2013 to 2014 in Shanghai presented a higher detection rate than the initial strategy. Nevertheless, the current contact investigation strategy identifies only a small percentage of TB disease patients. Given the high risk of TB disease and the low detection rate among contacts, the current strategy of contact investigation should be further improved. More attention should be paid to the identification of index cases, the diagnostic method, and the appropriate follow-up length.

## Abbreviations

AFB: Acid-fast bacillus
BALF: Bronchoalveolar lavage fluid
BCG: Bacillus Calmette-Guerin
BoDP&C-Moh: Bureau of Disease Prevention and Control under the Ministry of Health
CDC: Center for Disease Control and Prevention
CI: Confidence interval
DOTs: Directly Observed Treatment strategy
MDGs: Millennium Development Goals
PCR: Polymerase chain reaction
PPD: Purified protein derivative
PYs: Person-years
SD: Standard deviation
TB: Tuberculosis
